# Prognostic Value of the Platelet-to-Lymphocyte Ratio in Patients With Melanoma: A Meta-Analysis

**DOI:** 10.3389/fonc.2020.01116

**Published:** 2020-07-28

**Authors:** Feng Zhang, Weihong Gong

**Affiliations:** ^1^Department of Oncology, Linyi People's Hospital, Linyi, China; ^2^Department of Comprehensive Intervention, Linyi People's Hospital, Linyi, China

**Keywords:** platelet-to-lymphocyte ratio, meta-analysis, melanoma, prognostic factor, clinical use

## Abstract

**Background:** The prognostic role of the platelet-to-lymphocyte ratio (PLR) is controversial in patients with melanoma. Therefore, we performed a meta-analysis to assess the prognostic value of the PLR in patients with melanoma.

**Methods:** PubMed, Web of Science, Embase, Cochrane library, WanFang, and China National Knowledge Infrastructure were searched for eligible studies. Pooled hazard ratios (HRs) and 95% confidence intervals (CIs) were calculated to evaluate the association between the PLR and overall survival (OS) and progression-free survival (PFS).

**Results:** Nine studies with 2,396 patients were included in this meta-analysis. A high PLR was a predictor of shorter OS (HR = 1.67, 95% CI = 1.18–2.38, *p* = 0.004), but not PFS (HR = 1.53, 95% CI = 0.96–2.44, *p* = 0.075) in patients with melanoma. Subgroup analysis revealed that the PLR remained a significant prognostic indicator of both OS and PFS in patients with non-metastatic disease; the PLR cutoff value of <120 had a consistent prognostic value.

**Conclusions:** An increased PLR was associated with poor OS of patients with melanoma. Hence, we suggest that the preoperative PLR could be used to identify high-risk patients and provide information regarding the prognosis of patients with melanoma.

## Introduction

Melanoma is a highly aggressive skin cancer with an increasing incidence rate in Caucasians (1). In 2018, ~287,723 cases of melanoma were newly diagnosed, and 60,712 patients died of melanoma worldwide (2). In the United States, melanoma is the most rapidly increasing malignancy in men and the second most rapidly increasing cancer in women (after lung cancer) (3). Most newly diagnosed patients have localized disease and are curable with surgical excision as the primary treatment (3). For patients with advanced and metastatic stages of the disease, promising treatments include adjuvant and neoadjuvant immunotherapy using immune checkpoint inhibitors, stereotactic radiosurgery, and novel targeted therapies (4, 5). The prognosis of patients with stage I disease is excellent, with a 10-year overall survival (OS) rate of 94–98% (1). However, the prognosis of metastatic melanoma has not sustainably changed over the past several decades, with a 10-year OS rate of only 10–15% (1). Therefore, identifying simple, low-cost biomarkers is important for developing individualized treatments and evaluating the prognosis of patients.

Recent evidence has demonstrated that cancer-related inflammation plays a crucial role in the development of metastasis in patients with melanoma (6). Several inflammation-based parameters in blood have been explored as prognostic biomarkers for melanoma, including the neutrophil-to-lymphocyte ratio (NLR), platelet-to-lymphocyte ratio (PLR), systemic immune-inflammation index, and C-reactive protein (CRP) (7–9). Many studies have confirmed that the PLR is an independent prognostic factor for gastric cancer (10), bladder cancer (11), esophageal cancer (12), breast cancer (13), and non-small-cell lung cancer (14). A number of studies have also explored the prognostic significance of the PLR in patients with melanoma, although the results remain controversial (15–23). For example, some researchers reported that higher pretreatment PLR was a poor prognostic marker for patients with melanoma (16, 17, 19); whereas some other investigators found that there was a non-significant association between PLR and survival outcomes in melanoma (18, 23). Therefore, we performed a meta-analysis to systematically analyze the prognostic value of the PLR in patients with melanoma by using the currently available clinical evidence. We aimed to identify the prognostic significance of PLR in the survival outcomes of patients with melanoma through comparing the prognosis of patients with high and low PLR in prospective trials and retrospective cohort studies.

## Materials and Methods

### Search Strategy

The present meta-analysis was conducted according to the Preferred Reporting Items for Systematic Reviews and Meta-Analyses 2009 guidelines (24). We performed a comprehensive literature search of the following databases: PubMed, Web of Science, Embase, Cochrane library, WanFang, and China National Knowledge Infrastructure. The latest search was performed on May 8, 2020. The following search terms were used: “platelet to lymphocyte ratio,” “PLR,” “platelet–lymphocyte ratio,” “platelet lymphocyte ratio,” and “melanoma.” There were no year or language restrictions. In addition, the references of the papers were also manually checked for potential inclusions. As the study is a meta-analysis and does not involve the collection of samples, ethical approval is not necessary.

### Selection Criteria

The inclusion criteria for eligible studies were as follows: (1) the diagnosis of melanoma was histologically or pathologically confirmed; (2) the PLR values were measured before any treatment; (3) studies provided the data for the association between the PLR and survival outcomes including OS and/or progression-free survival (PFS); (4) the hazard ratio (HR) and 95% confidence interval (CI) for OS or PFS were reported or enough data were provided to calculate the HR and 95% CI; (5) a cutoff value of PLR was identified; and (6) the paper was published in English or Chinese. The exclusion criteria were as follows: (1) duplicate studies; (2) case reports, reviews, comments, and meeting abstracts; (3) non-human studies; and (4) insufficient data to calculate HRs and 95% CIs.

### Data Extraction

Two investigators (FZ and WG) independently extracted the information from eligible studies *via* a cross-check, and any disagreements were resolved by discussion. The following information was extracted from each included study: first author's name, year of publication, country of origin, patient age, study period, patient sex, sample size, PLR cutoff value, clinical stage, metastatic status, therapeutic strategy, follow-up, survival outcomes, HRs and 95% CIs for OS and PFS, and quality scores.

### Quality Assessment

The quality of the included studies was assessed by using the Newcastle–Ottawa Scale (NOS) (25). The NOS comprises three aspects: selection (0–4 stars), comparability (0–2 stars), and outcome (0–3 stars). The NOS score ranges from 0 to 9; studies with a NOS score ≥6 points are regarded as high-quality studies.

### Statistical Analysis

For studies assessing the OS and PFS, the HRs and 95% CIs were directly extracted if they were reported. Otherwise, the HRs and 95% CIs were calculated according to the methods described by Tierney et al. (26). The heterogeneity among studies was quantitatively evaluated by using the chi-squared Q test and the *I*^2^ metric statistic. If the *p*-value was < 0.10 on the Q test or if the *I*^2^ > 50%, indicative of significant heterogeneity, the random-effects model was applied. Otherwise, a fixed-effects model was selected. Subgroup analysis was conducted on the basis of sample size, disease status, and the PLR cutoff value. Meta-regression analysis was performed to identify the factors that may cause heterogeneity. Potential publication bias was estimated by using the Begg test (27) and the Egger test (28). All statistical analyses were performed by using Stata software, version 12.0 (Stata Corp., College Station, TX, USA). *P* < 0.05 were considered statistically significant.

## Results

### Search Results and the Description of the Studies

**Figure 1:**
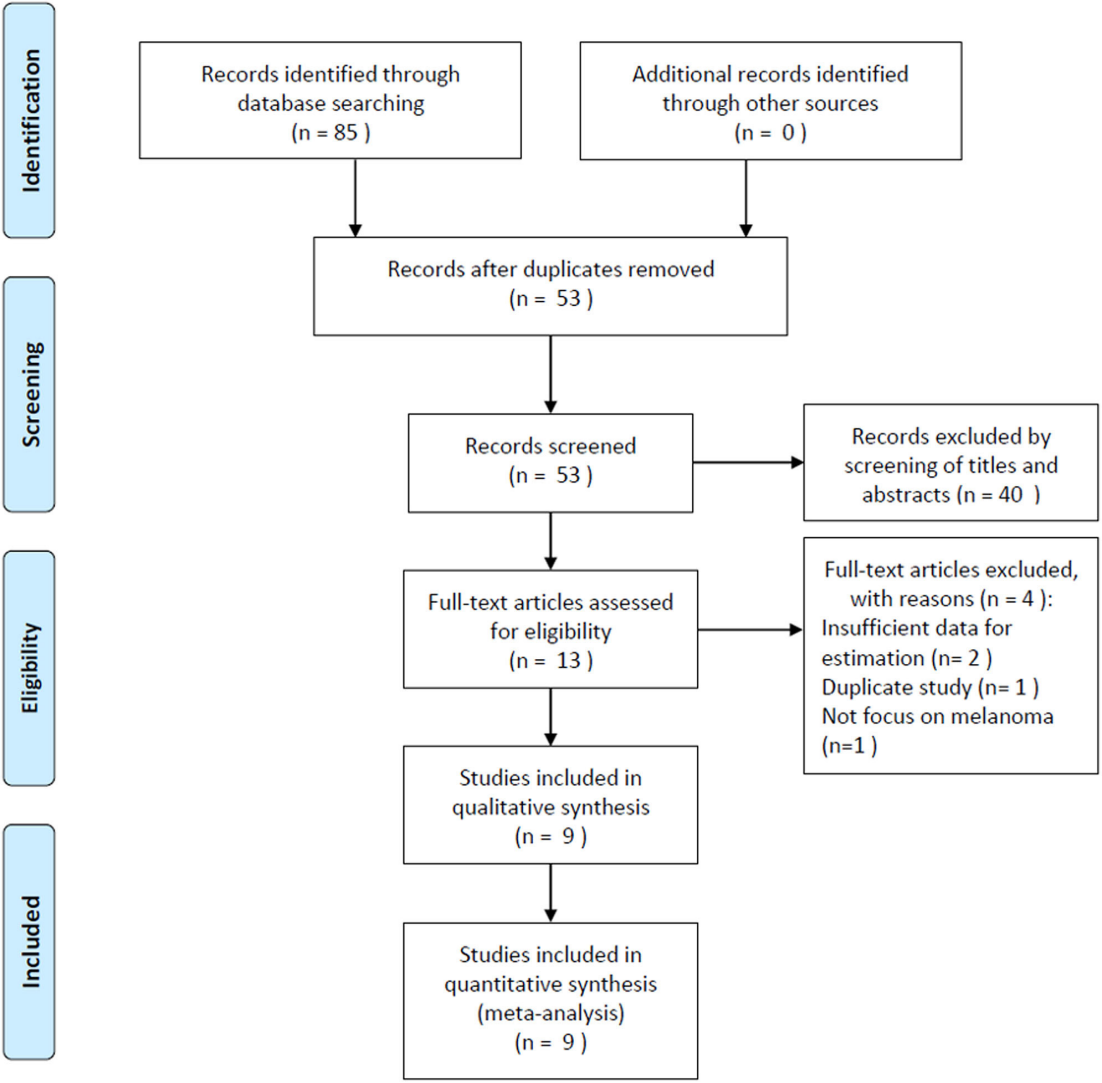
The flowchart of study selection procedure in the meta-analysis.

The initial literature search yielded 85 records, and 53 studies were further scanned after the removal of duplicates. Then, 40 studies were excluded after screening the title and abstract, following which, 13 studies were evaluated by examining the full text. Subsequently, four studies were eliminated owing to the following reasons: two articles had insufficient data, one was a duplicate study with overlapping patients, and one did not focus on melanoma. Finally, nine studies (15–23) were included in this meta-analysis. Figure 1 illustrates the searching and selection process of the included studies. The included studies were published between 2016 and 2019. The included studies encompassed a total of 2,396 patients, ranging from 21 to 1,351 patients, with a median value of 140. The studies were conducted in four countries, including China (*n* = 6) (16, 17, 19, 21–23), Canada (*n* = 1) (15), Japan (*n* = 1) (18), and the UK (*n* = 1) (20). Seven studies were published in English (15, 16, 18–20, 22, 23), and two were in Chinese (17, 21). Eight studies with 2,341 patients (15–22) provided the data for OS, and five studies with 1,892 patients (15, 16, 20, 22, 23) presented the prognostic value of the PLR considering PFS. The cutoff values of the PLR ranged from 99 to 206; the median value was 120. Patients who underwent surgery were included in three studies (17, 20, 21), patients who received mixed treatment were included in two studies (19, 22), and patients receiving anti-cytotoxic T lymphocyte-associated protein (CTLA)-4 (15), interferon (IFN)-α-2b (16), anti-PD-1 (18), or chemotherapy (23) were included in one study each. The main characteristics of the included studies are shown in Table 1. The NOS scores for all the studies ranged from 6 to 9, indicating that all the included studies were of high quality.

**Table 1:** Characteristics of the included studies in this meta-analysis.

**Author**	**Year**	**Country**	**Sample size**	**Age median (range)**	**Sex (male/female)**	**Disease status**	**Study period**	**Stage**	**Treatment**	**Follow-up (months) Median (range)**	**Cutoff value**	**NOS score**	**Survival analysis**
Khoja	2016	Canada	183	58 (24–89)	115/68	Metastatic	2008–2015	IV	Anti-CTLA-4	7.5 (0.3–49.5)	182	8	OS, PFS
Yu	2017	China	263	NR	NR	Metastatic	2010–2016	IV	IFN-α-2b	To October 2016	129	6	OS, PFS
Cao	2018	China	120	57 (19–86)	67/53	Non metastatic	2007–2012	I-III	Surgery	93	99	7	OS
Minowa	2018	Japan	21	74 (34–91)	11/10	Metastatic	2014–2017	IV	Anti-PD-1	NR	159	6	OS
Qi	2018	China	140	56.4 (22–81)	69/71	Mixed	2010–2015	I-IV	Mixed	21.5 (1–80)	120	8	OS
Wade	2018	UK	1351	NR	678/673	Non metastatic	2006–2016	I-III	Surgery	45.6 (3–128.4)	100	9	OS, PFS
Wang	2019	China	223	55.6 (18–85)	136/87	Non metastatic	2005–2015	I-III	Surgery	To May 2018	113.6	7	OS
Wang	2019	China	40	58	14/26	Mixed	2010–2017	I-IV	Mixed	47.8 (7.8–88.9)	118.7	8	OS, PFS
Yang	2019	China	55	58 (38–75)	23/32	Metastatic	2013–2018	III-IV	Chemotherapy	19.2 (2.9–37.1)	206	7	PFS

*OS, overall survival; PFS, progression-free survival; NR, not reported; IFN-α-2b, interferon alfa-2b; NOS, Newcastle–Ottawa Scale*.

### The Association Between the Platelet-to-Lymphocyte Ratio and Overall Survival in Patients With Melanoma

**Figure 2:**
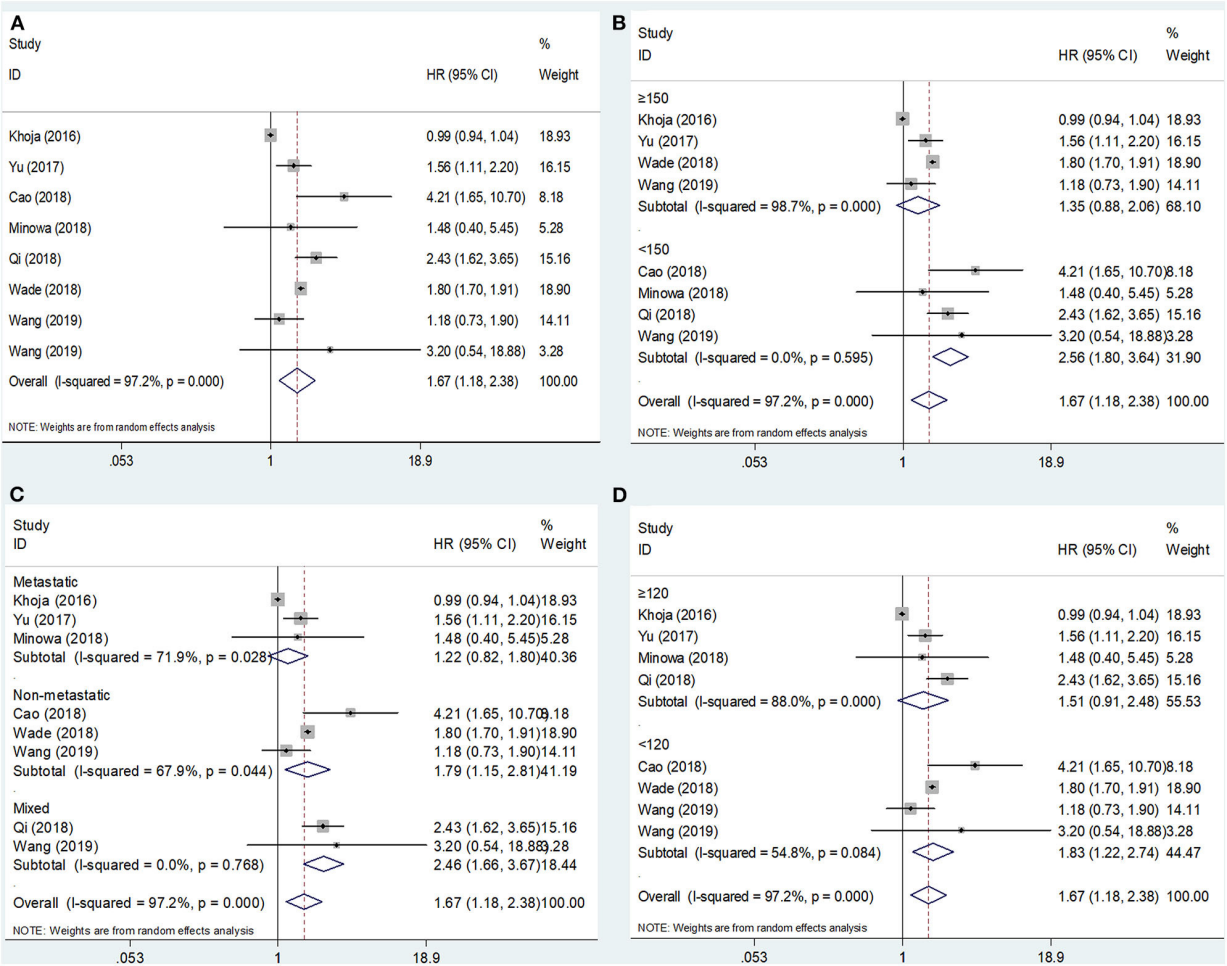
Forest plot hazard ratio (HR) for the correlation between elevated platelet-to-lymphocyte ratio (PLR) and overall survival in melanoma. **(A)** For the total patients' group; **(B)** Subgroup analysis stratified by sample size; **(C)** Subgroup analysis stratified by disease status; and **(D)** Subgroup analysis stratified by the cutoff value of PLR. All analyses were performed using a random-effects model.

The association between the PLR and OS was reported in eight studies including 2,341 patients (15–22). The HRs and 95% CIs were extracted and combined by using a random-effects model because of significant heterogeneity (*I*^2^ = 97.2%, *p* < 0.001; Figure 2A; Table 2). The pooled HR and 95% CI were 1.67 and 1.18–2.38, respectively (*p* = 0.004), suggesting that a high PLR was correlated with poor OS. Then, subgroup analysis was conducted to determine the source of heterogeneity. Regarding the sample size, an elevated PLR was associated with poor OS of studies enrolling cases <150 (HR = 2.56, 95% CI = 1.80–3.64, *p* < 0.001) but not of studies with a sample size ≥150 (HR = 1.35, 95% CI = 0.88–2.06, *p* = 0.166; Figure 2B; Table 2). Considering the disease status, a high PLR predicted poor OS in patients with non-metastatic disease (HR = 1.79, 95% CI = 1.15–2.81, *p* = 0.011) and those with mixed status (HR = 2.46, 95% CI = 1.66–3.67, *p* < 0.001), but not in those with metastatic disease (HR = 1.22, 95% CI = 0.82–1.80, *p* = 0.325; Figure 2C; Table 2). Regarding the cutoff value of the PLR, a PLR of < 120 remained a significant prognostic factor for OS (HR = 1.83, 95% CI = 1.22–2.74, *p* = 0.003), whereas a PLR of ≥120 was not significant (HR = 1.51, 95% CI = 0.91–2.48, *p* = 0.108; Figure 2D; Table 2).

**Table 2:** The subgroup analysis between PLR and the prognosis of patients with melanoma.

**Variables**	**No. of studies**	**No. of patients**	**Effects model**	**HR (95% CI)**	***p***	**Heterogeneity** ***I***^****2****^**(%)** ***P***		**Meta-regression *p***
**OS**								
Total	8	2341	Random	1.67 (1.18–2.38)	0.004	97.2	<0.001	
Sample size								0.066
<150	4	321	Fixed	2.56 (1.80–3.64)	<0.001	0	0.595	
≥150	4	2020	Random	1.35 (0.88–2.06)	0.166	98.7	<0.001	
Disease status								0.105
Non-metastatic	3	1694	Random	1.79 (1.15–2.81)	0.011	67.9	0.044	
Metastatic	3	467	Random	1.22 (0.82–1.80)	0.325	71.9	0.028	
Mixed	2	180	Fixed	2.46 (1.66–3.67)	<0.001	0	0.768	
PLR cutoff value								0.516
<120	4	607	Random	1.83 (1.22–2.74)	0.003	54.8	0.084	
≥120	4	1734	Random	1.51 (0.91–2.48)	0.108	88.0	<0.001	
**PFS**								
Total	5	1892	Random	1.53 (0.96–2.44)	0.075	95.5	<0.001	
Sample size								0.555
<150	2	95	Fixed	1.92 (1.01–3.63)	0.045	0	0.979	
≥150	3	1797	Random	1.41 (0.82–2.45)	0.216	97.7	<0.001	
Disease status								0.274
Non-metastatic	1	1351	-	1.90 (1.67–2.16)	<0.001	-	-	
Metastatic	3	501	Random	1.27 (0.82–1.96)	0.288	54.2	0.112	
Mixed	1	40	-	1.95 (0.48–8.02)	0.353	-	-	
PLR cut-off value								0.266
<120	2	1391	Fixed	1.90 (1.67–2.16)	<0.001	0	0.970	
≥120	3	501	Random	1.27 (0.82–1.96)	0.288	54.2	0.112	

*OS, overall survival; PFS, progression-free survival; PLR, plate-to-lymphocyte ratio*.

### The Association Between the Platelet-to-Lymphocyte Ratio and Progression-Free Survival in Patients With Melanoma

**Figure 3:**
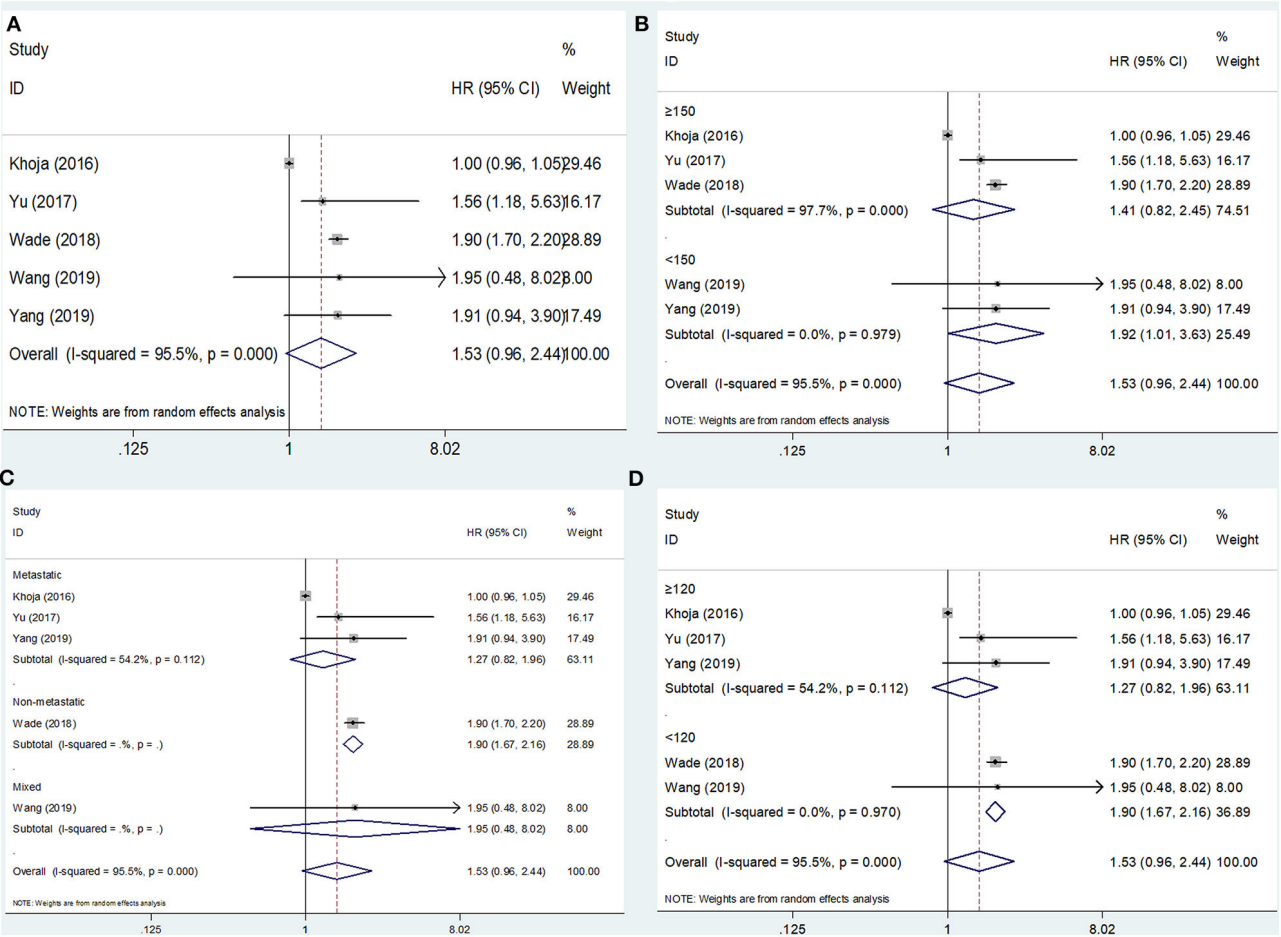
Forest plot hazard ratio (HR) for the correlation between elevated platelet-to-lymphocyte ratio (PLR) and progression-free survival in melanoma. **(A)** For the total patients' group; **(B)** Subgroup analysis stratified by sample size; **(C)** Subgroup analysis stratified by disease status; and **(D)** Subgroup analysis stratified by cutoff value of PLR. All analyses were performed using a random-effects model.

The PFS outcomes from five studies comprising 1,892 patients were analyzed (15, 16, 20, 22, 23). The heterogeneity was significant (*I*^2^ = 95.5%, *p* < 0.001); therefore, a random-effects model was applied. The pooled results suggested that an elevated PLR was not significantly correlated with PFS (HR = 1.53, 95% CI = 0.96–2.44, *p* = 0.075; Figure 3A; Table 2). On subgroup analysis, the pooled data showed that a high PLR was correlated with poor PFS in studies with a sample size <150 (HR = 1.92, 95% CI = 1.01–3.63, *p* = 0.045; Figure 3B; Table 2) and in those with non-metastatic disease (HR = 1.90, 95% CI = 1.67–2.16, *p* < 0.001; Figure 3C; Table 2), as well as when the cutoff value of the PLR was <120 (HR = 1.90, 95% CI = 1.67–2.16, *p* < 0.001; Figure 3D; Table 2). However, the PLR was not associated with the PFS in studies with a sample size ≥150 and in those with metastatic and mixed disease, as well as when the cutoff value of the PLR was ≥120 (Table 2).

### Meta-Regression Analysis

We performed a meta-regression analysis to determine the factor (sample size, disease status, or cutoff value) that may cause heterogeneity. As shown in Table 2, the data suggested that sample size, disease status, and the cutoff value did not result in significant heterogeneity in OS and PFS (*p* > 0.05 for all).

### Publication Bias

**Figure 4:**
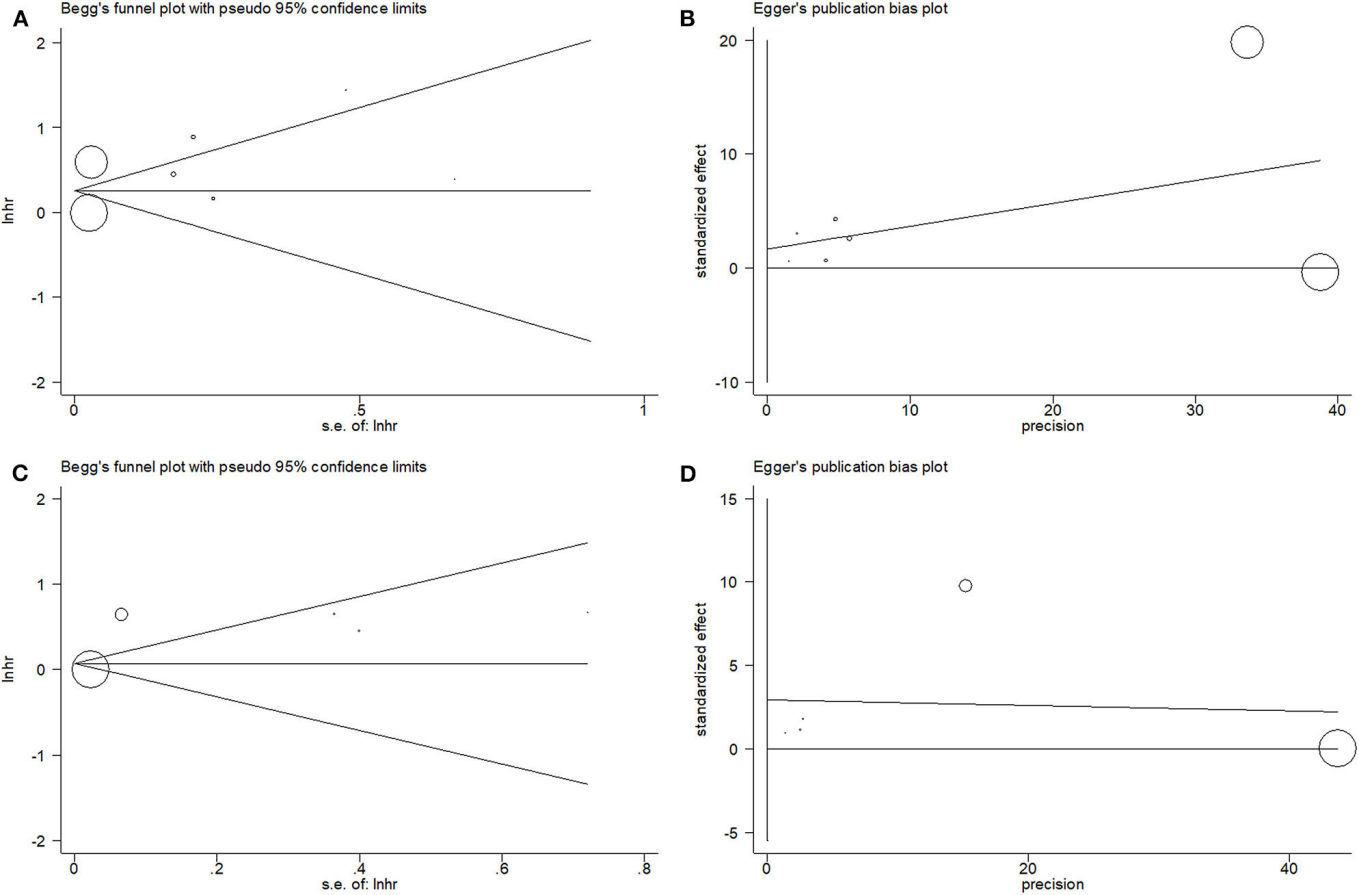
Begg's funnel plot and Egger's test to evaluate publication bias. **(A)** Begg's test for overall survival (OS); **(B)** Egger's test for OS; **(C)** Begg's test for progression-free survival (PFS); and **(D)** Egger's test for PFS.

For the meta-analysis with OS and PFS, the publication bias was assessed by using the Begg test and the Egger test. For OS, the *p*-value on the Begg test was 0.902 (Figure 4A), and the *p*-value on the Egger test was 0.575 (Figure 4B). For PFS, the *p*-value on the Begg test was 0.806 (Figure 4C), and the *p*-value on the Egger test was 0.350 (Figure 4D). The results revealed that there was no significant publication bias in the current meta-analysis.

## Discussion

Inflammation responses play an important role in tumor initiation, progression, and metastasis (29). Many inflammatory indicators—derived from the peripheral blood—and cancer-related inflammatory cells have been investigated as prognostic factors for melanoma, such as the NLR, PLR, CRP, and tumor-infiltrating lymphocytes (TILs) (7, 30, 31). Although the prognostic utility of the PLR has been evaluated in patients with melanoma, the results are still controversial (15–17, 19, 20, 22). Therefore, in the current meta-analysis, we pooled the data from nine studies and found that an elevated PLR was associated with poor OS but not PFS of patients with melanoma. The results of subgroup analysis indicated that an elevated PLR predicted poor OS in most subpopulations. In addition, the PLR remained a significant prognostic indicator for patients with non-metastatic disease; moreover, the PLR cutoff value of <120 had a consistent prognostic value. Those findings suggested that the PLR was more sensitive regarding the prognosis of patients with localized tumors. Furthermore, a PLR <120 showed a more potent prognostic efficacy for the prognostication of melanoma. Taken together, the results of our study demonstrated that a high PLR was associated with poor OS of patients with melanoma. Moreover, our results suggested that the PLR could serve as a promising prognostic parameter for melanoma. To the best of our knowledge, the current study is the first meta-analysis to identify the association between the PLR and survival outcomes of patients with melanoma.

Cancer-related systemic inflammation plays an essential role in every step of tumor development, including proliferation, angiogenesis, and metastasis (32). A high PLR is the result of an increase in platelets and/or a low lymphocyte count. Platelets are critical sources of cytokines and can facilitate tumor progression by sustaining proliferative signaling (33), support tumor growth and metastasis (34), and protect cancer cells from apoptosis (35) through their secretion of interleukin (IL)-6, tumor necrosis factor (TNF)-α, and platelet-derived growth factor (PDGF), among other factors. In addition, platelets can support tumor cells in evading the immune system of the host (36). In contrast, lymphocytes, especially TILs, play a vital role in T cell-mediated antitumor response (37). Lymphocytes exert an antitumor effect *via* the induction of cytotoxic cell death as well as the suppression of tumor proliferation through the secretion of IL-4, IL-5, and TNF-β (38). Many studies showed that a low lymphocyte count was correlated with poor prognoses of multiple malignancies (39–41). Therefore, a high PLR could be an indicator of the low antitumor capacity and thus predict poor prognosis.

Many studies have investigated the prognostic significance of the PLR in human tumors through meta-analyses (42, 43). A recent meta-analysis including 28 studies with 15,617 patients indicated that an elevated PLR was associated with poor OS of patients with gastric cancer (44). Another meta-analysis also revealed that the pretreatment PLR was a simple, promising prognostic indicator of OS and PFS of patients with ovarian and cervical cancers (45). Furthermore, a high PLR was associated with poor prognosis in patients with nasopharyngeal carcinoma (46). In the current meta-analysis, we identified the PLR as a prognostic factor for OS but not for PFS in patients with melanoma; the non-significant prognostic value for PFS may be the result of the limited studies included. The observations of the current study are in accordance with the findings for bladder cancer (47) and diffuse large B-cell lymphoma (48). Nevertheless, the prognostic value of PLR for PFS needs to be further explored in large-scale prospective trials.

This study has several limitations. First, the sample size was relatively small; only nine studies with 2,396 patients were included in this meta-analysis. For PFS analysis, only five studies were eligible, and the association between the PLR and clinicopathological features of melanoma could not be analyzed because of insufficient data. As the sample size was limited, the persuasiveness of the results may be compromised. Second, the cutoff value of the PLR was different among the included studies. A standard value for a high PLR is needed to facilitate the wide application of the PLR in clinical practice. Third, the obvious heterogeneity of the included studies cannot be ignored. Although we adopted the random-effects model to analyze data when the heterogeneity was significant, the heterogeneity still existed. We also conducted a meta-regression analysis, wherein the results suggested that sample size, disease status, and the cutoff value did not result in significant heterogeneity. Accordingly, the source of the heterogeneity could be the retrospective nature of the included studies, selection bias of patients, and diverse treatment methods. Therefore, we suggest that well-designed, prospective trails be conducted in the future to verify the results of the current meta-analysis.

In summary, the results of the present meta-analysis demonstrated that an elevated PLR was associated with poor OS of patients with melanoma. Moreover, the PLR remained a significant prognostic indicator for patients with non-metastatic disease; in addition, the PLR cutoff value of <120 had a consistent prognostic value. We recommend that the pretreatment PLR be used to identify high-risk patients and determine the prognosis of patients with melanoma.

## Data Availability Statement

All datasets generated for this study are included in the article/supplementary material.

## Author Contributions

FZ and WG performed the retrieval of data and took part in the statistical analysis for meta-analysis. WG drafted and revised the manuscript. All authors read and approved the final manuscript.

## Conflict of Interest

The authors declare that the research was conducted in the absence of any commercial or financial relationships that could be construed as a potential conflict of interest.
